# Commentary: One-year quality of life among post-hospitalization COVID-19 patients

**DOI:** 10.3389/fpubh.2024.1417068

**Published:** 2024-07-03

**Authors:** Josef Finsterer

**Affiliations:** Neurology & Neurophysiology Center, Vienna, Austria

**Keywords:** SARS-CoV-2 infection, quality of life, questionnaire, outcome, SARS-CoV-2 vaccination

## Introduction

There is growing evidence that SARS-CoV-2 infections (SC2Is) can be complicated by chronic conditions that can last for weeks or months. For didactic reasons, these enduring complications are termed post-COVID syndrome (PCS) if they last for < 12 weeks and long-COVID syndrome (LCS) if they last for >12 weeks. Although more and more studies are being conducted on these topics, the need for further discussion remains, as the following publication shows.

## Study of interest

The interesting study by Perez Catalan et al. focused on the quality of life (QoL) 1 year after a SC2I in 486 patients through telephone interviews using the SF-36 QoL questionnaire (1). While the findings are compelling, certain aspects of the study warrant further discussion.

## Discussion

The first point is that telephone interviews have several disadvantages. First, it cannot be determined whether the person called is indeed the patient in question. Second, there is no way to verify that the responses accurately reflect the actual events. Third, telephone interviews do not allow for the request of additional tests to generate new data.

The second point is the SF-36 questionnaire itself. The questions are not tailored specifically to SC2Is, focusing instead on general wellbeing. To effectively assess the outcome of SC2Is after 1 year, it would be desirable to ask specific questions about SC2Is and obtain detailed information on its common symptoms.

A third point is that within 1 year of suffering from SC2Is, patients might develop multiple diseases, receive new medications, or undergo various medical procedures. Therefore, it is crucial to inquire about any new comorbidities, medications, or treatments that have been introduced since the SC2I to fully assess their current health status and the ongoing impacts of the infection.

A fourth point is that the impact of the SARS-CoV-2 vaccination (SC2V) on QoL during the follow-up period was not discussed. In how many cases was the SC2V tolerated without side effects, and in how many patients was the SC2V complicated by adverse reactions? Sometimes, it may not be the SC2I itself but the vaccination that could impair QoL. Therefore, it would be beneficial to further address the potential impact of SC2V on QoL during the follow-up period. This discussion should include comprehensive and appropriate references on infection prevention and public health guidance, including the occurrence of any adverse reactions. These factors could significantly influence patients' health perceptions and outcomes. A more comprehensive understanding of the factors influencing PCS/LCS, along with the judicious use of artificial intelligence and machine learning algorithms, can facilitate real-time monitoring, sophisticated data interpretation, and agile decision-making in relevant and responsive National Immunization Programs (NIPs).

To assess QoL 1 year after a SC2I, on-site examinations are preferable to telephone questionnaires. For patients complaining of long-lasting COVID symptoms, further investigation should be planned to determine whether these symptoms are directly related to previous SC2I. Future research should prioritize face-to-face assessments, utilize targeted questionnaires, gather comprehensive medical histories, and conduct detailed analyses of vaccine effectiveness to provide a more accurate and thorough assessment of PCS and LCS.

## Author contributions

JF: Conceptualization, Investigation, Methodology, Software, Supervision, Writing – original draft, Writing – review & editing.
